# Gene exchange between Neisseria meningitidis and Neisseria gonorrhoeae

**DOI:** 10.1099/mgen.0.001623

**Published:** 2026-01-19

**Authors:** Sebastiaan J. van Hal, Frances Jenkins, Tiffany R. Hogan, Sanghamitra Ray, Ratan L. Kundu, Helen S. Marshall, Rory Bowden, Monica M. Lahra

**Affiliations:** 1 Department of Infectious Diseases and Microbiology, NSW Health Pathology, Royal Prince Alfred Hospital, Sydney, NSW 2050, Australia; 2 Central Clinical School, University of Sydney, Sydney, NSW 2006, Australia; 3 World Health Organization Collaborating Centre for STI and AMR, New South Wales Health Pathology Microbiology, The Prince of Wales Hospital, Randwick, NSW 2031, Australia; 4 Vaccinology and Immunology Research Trials Unit, Women’s and Children’s Health Network, Adelaide, South Australia, Australia; 5 Adelaide Medical School and Robinson Research Institute, Adelaide University, Adelaide, South Australia, Australia; 6 Walter and Eliza Hall Institute of Medical Research, Parkville, VIC 3052, Australia; 7 Department of Medical Biology, University of Melbourne, Parkville, VIC 3052, Australia; 8 Faculty of Medicine, The University of New South Wales, Kensington, NSW 2052, Australia

**Keywords:** antimicrobial resistance, co-carriage, genetic exchange, *Neisseria gonorrhoeae*, *Neisseria meningitidis*, pan-genome analysis, phylogenetics

## Abstract

Genetic exchange between *Neisseria meningitidis* (NM) and *Neisseria gonorrhoeae* (NG) has not been well studied. This study aimed to investigate evidence of genetic exchanges between these two species. All coincident paired NM and NG isolates cultured from pharyngeal swabs collected from a sexual health clinic in Sydney in 2021 underwent whole-genome sequencing. A gene-by-gene analysis of the 47 NM–NG pairs identified 184 instances where the ancestry of the gene revealed intermixing between the two species. Incorporating the gene phylogenies demonstrated that these events occurred across a wide range of timeframes. At the nucleotide level, 91 genes were found where paired isolates harboured identical sequences. Notably, one instance of unequivocal recent gene transfer events between the paired pharynx isolates was observed. This work provides new insights into the evolutionary dynamics of these bacteria and highlights the importance of genetic exchange in populations with high rates of pharyngeal gonorrhoea. The clinical implications of such exchanges call for continued vigilance and research to address the challenges posed by these bacteria.

**Impact Statement**

Recombination among *Neisseria* species is well recognized, with evidence of DNA exchanges involving commensal *Neisseria* and the pathogenic species *Neisseria meningitidis* or *Neisseria gonorrhoeae*. Direct horizontal gene transfer between *N. meningitidis* and *N. gonorrhoeae* is rare, partly because these pathogens typically occupy distinct ecological niches. In individuals concurrently carrying both pathogenic *Neisseria* species in their pharynx, we were able to identify clear evidence of gene exchange, including a confirmed instance of very recent transfer, between both species. These findings demonstrate that interactions between these two pathogenic *Neisseria* species are more common and temporally diverse than previously appreciated. This study highlights the complexity of exchange events between *Neisseria* species and validates concerns about sharing of antimicrobial resistance determinants particularly in populations with high rates of pharyngeal gonorrhoea.

## Data Summary

The sequence reads for all isolates are available from NCBI under project number PRJNA1046565. For additional and individual isolate details, see the Supplementary Data File.

## Introduction

*Neisseria meningitidis* (NM) and *Neisseria gonorrhoeae* (NG) are closely related, yet ecologically and pathogenetically distinct, species. NM is primarily a commensal of the human oropharynx, with carriage rates of ~10% in adolescents [1,2]. Carriage may lead to invasive infections that, although rare, are associated with a case fatality rate of up to 50% [3]. In contrast, NG is always considered a pathogen and predominantly transmitted through sexual contact [4]. *NG* infections typically manifest in the mucosa of the urethra and reproductive tract (cervix, fallopian tubes and uterus), as well as the rectum, pharynx and conjunctiva [5,6].

Whilst NM and NG share a common ancestral species [7], the timing of their separation to distinct species remains unknown and obscured by the continuing exchange of genetic material [8]. Despite this genetic exchange and general genomic and phenotypic similarities, their patterns of genetic diversity and evolutionary trajectories are quite different. NM maintains considerable genetic diversity in the form of genetically well-defined clusters, consistent with a large, stable effective population size [9,10]. In contrast, NG populations tend to comprise a single dominant clone with low sequence diversity, reflecting a smaller effective population size associated with clustered transmissions. The latter pattern likely originates in the species’ emergence from a single evolutionary event, driving a niche change from the pharynx to the urogenital tract, and an ecological transition from commensal to pathogen [6,11].

Multiple *Neisseria species*, including NM and NG, share similar mucosal niches in humans, which promotes the exchange of genetic material within the genus [12]. *Intra*-species exchange events (for example, amongst NM *lineages*) continue to shape overall *species* diversity and are well documented and considered to be common [10]. *Inter*-species events between pathogenic and non-pathogenic commensal pharyngeal *Neisseria* spp. such as *N. lactamica*, *N. cinerea* and *N. subflava* are less common [13,14]. However, these events remain significant because they can result in a species acquiring a new phenotype, for example, through the transfer of antimicrobial resistance (AMR) determinants, as described with NG [15].

Understanding the tempo and modes of genetic exchange between pathogens, especially in the context of changing epidemiology, is important in devising disease control and treatment strategies and understanding new risks to health. Identifying isolates linked by recent genetic transfer is significant because it may help us understand the conditions driving sequence exchange and the kinds of molecular events involved. To our knowledge, genetic exchange between colonizing NM and NG infections has not been explored systematically.

In this study, we searched for genetic transfer between NM and NG isolates. We examined consecutive clinical pharyngeal swabs from high-incidence populations to determine the rates of NM and NG co-carriage on bacterial culture. We then sequenced a single isolate of each species from each patient and looked for evidence of recent inter-species transfer of genetic material.

## Methods

### Isolate collection

Consecutive pharyngeal swabs from patients attending an urban sexual health clinic in Sydney, Australia, between 1 January 2021 and 31 December 2021, routinely collected for either diagnostic (symptomatic) or screening (asymptomatic) *NG* testing. All positive NG samples were examined for co-carriage of NM. A total of 47 patients were identified with NG infection and coincident NM in the pharynx. Isolates were identified by MALDI-TOF, and identification of NM was confirmed as required using molecular targets. Susceptibilities were performed and reported on the isolates using the Clinical Laboratory Standards Institute methodology. Single colonies of the NM and NG isolates in the NG–NM pairs were sent for Illumina short-read sequencing.

Patients’ meningococcal vaccination status was unknown.

### Sequencing and genomic analysis

Following DNA extraction from a single colony using the EZ1 Advanced XL (Qiagen, Hilden, Germany), DNA libraries were generated using an Illumina DNA prep kit (Illumina, San Diego, CA, USA) and sequenced on the Illumina MiSeq platform according to the manufacturer’s instructions aiming for a target sequencing depth of ≥20×, a >90% k-mer match with the observed species and a minimum Phred quality score of 30 across the obtained read(s). Reads were first trimmed using fastp (v.0.22.0) [16] prior to generating assemblies using SPAdes (v3.15.3) [17] and removing contigs <1,000 bp in length.

The assemblies were first annotated with Prokka (v.1.14.6) [18] with the pan-genome constructed using Panaroo (v.1.2.8) [19] which implements a graph-based approach allowing for correction of annotation errors between genomes. Additional typing was performed using the assemblies and included *in silico* MLST, NG-MAST, NG-STAR and NM-serogrouping with isolate resistomes determined using AMRFinder (v.3.10.20) [20].

### Inter-species gene relationships at the phylogenetic and sequence levels

To investigate inter-species transfer events, a gene-by-gene analysis approach was taken.

Initially, individual gene trees were generated using iqtree2 (v.2.2.0.3) [21] substitution model (GTR+F+G4) with gene species relationships inferred from midpoint-rooted maximum likelihood phylogenies. Tree topologies were classified into three distinct patterns. The first pattern demonstrated clear species-level clustering without any evidence of mixing between species. The second pattern displayed overall species-level clustering but with interspersed sequences from the alternate species. The third pattern showed complete intermixing of sequences between species. These observed phylogenetic patterns remained the same when trees were not rooted.

For genes with at least one identical sequence within a pair, attempts were made to find the largest continuous sequence shared between pairs. Collinear blocks were extracted from isolate *de novo*-assembled contigs, provided that the sequence fragment met the following criteria: (i) consisted of at least two genes with intergenic region(s); (ii) gene synteny and gene orientation were identical across isolates; and (iii) the sequence block was not split by contig boundaries. The boundaries of collinear blocks were then used to obtain the same sequence region from all other isolates. Contigs with breaks in collinearity were excluded from the analysis.

Following alignment, using clustal Omega v.1.2.3 of the collinear blocks, evidence of exchanges was examined using fastGEAR [22,23]. Only events with a log(Bayes factor) >5, corresponding to ~150-fold marginal likelihood favouring a recombination event, were considered significant. Donor and recipient species were labelled based on the majority species membership of the inferred lineages.

### Statistics and calculations

Comparison between groups was performed using the chi-squared test in R. To calculate the probability of an event occurring during the co-carriage event, Bayes’ theorem was used and calculated using the equation:

$P\left(Co|sharedallele\right)=\frac{1*P\_co}{1*P\_co+P\_random*(1-P\_co)}$ ; where P_Co and P_random equate to the co-carriage and random probability of a shared allele within a pair. The likely probabilities of finding an exact allele randomly within a *Neisseria* species were derived from the frequency distributions obtained through PubMLST [24].

## Results

During the study period, 423 swabs were collected, of which 162 (162 out of 438, 38%) were positive for NG in this population. Of these, 47 out of 162 were also positive for NM (NG–NM pairs) and represented a 29% probability of co-carriage or P(C).

### Genotyping information

MLST, serogroup, clonal complex and resistomes are provided for the 47 NG–NM paired isolates (Supplementary Table, available in the online Supplementary Material). The 47 *NG* isolates were distributed among 10 MLST types and had resistance profiles consistent with those observed in Australia. Phenotypic resistance to penicillin (MIC >1 mg l^−1^) was observed in 15 NG isolates, all of which harboured resistance-associated mutations in the *mtr* and/or *porB* genes, as well as a plasmid-borne beta-lactamase (blaTEM-1) in all 12 NG isolates. Numerous *penA* mutations were detected, associated with a mosaic penA allele rather than ceftriaxone resistance. Azithromycin resistance was observed in one isolate. Quinolone resistance (MIC >0.5 mg l^−1^) in conjunction with *gyrA* and *parC* mutations was detected in 39 isolates. In contrast, the NM isolates fell into 23 sequence types with no antibiotic resistance detected. Amongst the NM isolates, 19 out of 47 (40%) were encapsulated (serogroups: B=15; X=2; W=1; E=1) with 6 of the 15 serogroup B isolate sequence types (cc-213; *n*=1 and ST-5662; *n*=5) similar to circulating invasive isolates in Australia in 2021 [25].

### Sharing of genes at species level

We first assessed patterns of sharing of protein-coding sequences at the species level in the *NG–NM* pairs, undertaking a pan-genome analysis [19]. A total of 3,297 genes were detected, of which 1,874 were shared between species, while 308 and 1,115 genes were restricted to NM and NG isolates, respectively. The number of genes present was similar in NM (median 1,974, range 1,870–2,132 genes) and *NG* (median 2,013, range 1,932–2,056) isolates. Of the 1,874 genes shared between species, 1,495 were present in at least 40 isolates from each species (Fig. S1).

**Fig. 1.**
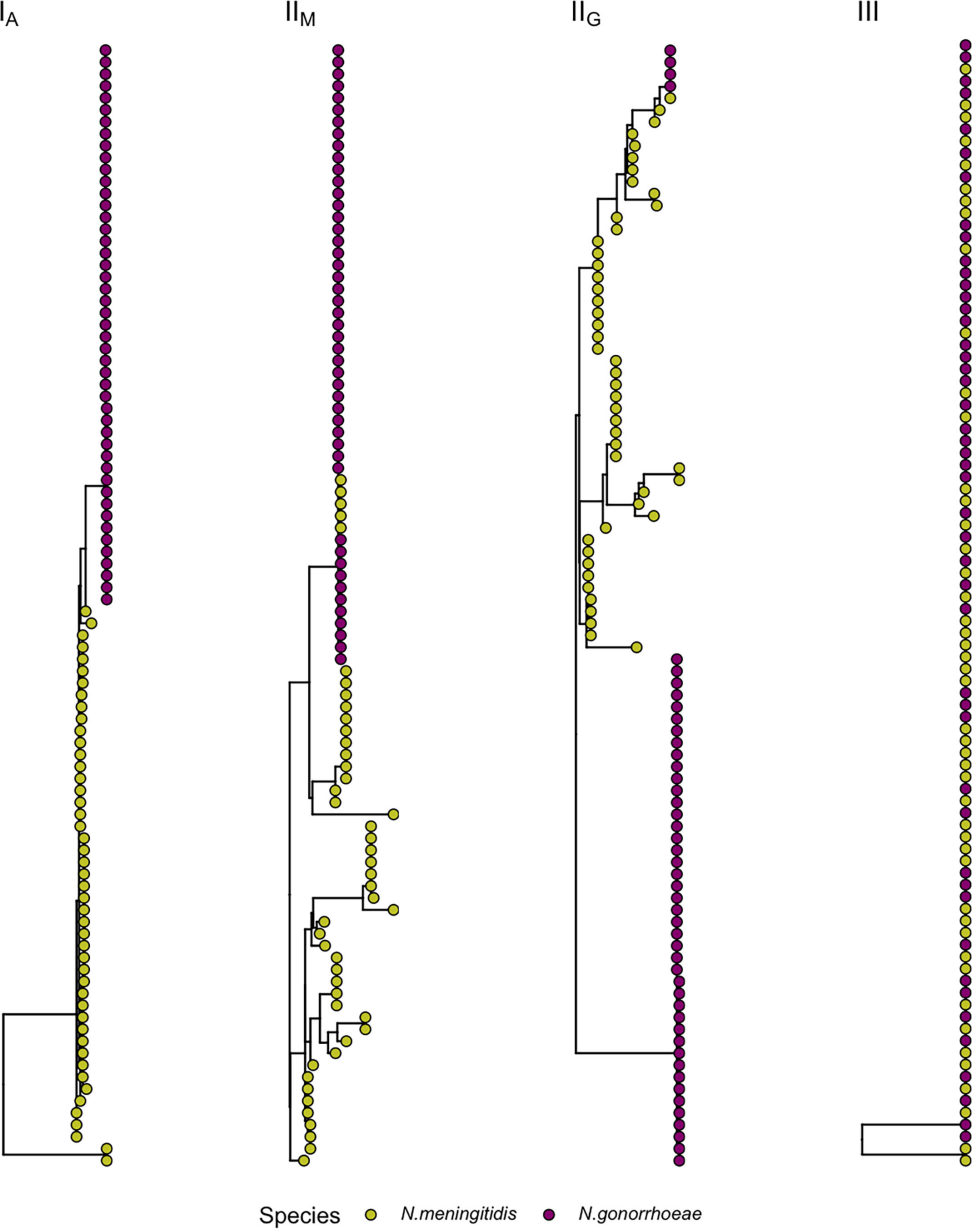
Phylogenetic gene patterns and shared gene lineages. Representative phylogenetic gene trees revealed three distinct patterns in the NG–NM pairs: (i) species-level separation with (I_A_) or without (not shown) a shared common gene ancestry; (ii) sequences from one species clustering with another, indicating gene fragment exchange. This includes an NG fragment exchange with an NM recipient (G*→*M) resulting in pattern II_M_ or an NM fragment exchange with an NG recipient (M*→*G) resulting in pattern II_G_; and (iii) a ‘mixed’ pattern with the two species sharing the same sequence distribution.

Patterns of gene ancestry were examined after generating individual-gene phylogenies for the 1,495 genes. The majority, 1,291 (86.4%), exhibited a clear species-level separation (pattern I_A_ in Fig. 1). In 184 (12.3%), phylogenies revealed sequences characteristic of 1 species clustering amongst isolates of the other species (patterns II_M_ and II_G_ in Fig. 1). In the remaining trees (*n*=20; 1.3%), a complete intermixing of sequences between species was observed (pattern III in Fig. 1). These genes were found to have a roughly equal carriage prevalence of ~50% in both species [24] and either originated external to the population (e.g. *maf* adhesin genes associated with genomic islands) or encoded highly conserved (e.g. ribosomal) proteins.

For the 184 genes where 1 species clustered with another, identified alleles were skewed to 1 species in PubMLST. When NG sequences clustered with NM, the identified allele within the NG isolates was associated with NM genomes greater than 84.5% of the time for an average of ~5,600 genomes. Conversely, shared NG alleles within NM were found in NG genomes 83% of the time for an average of ~11,700 genomes (Supplementary Table). These proportions of carriage and tree topologies strongly suggested a gene allele acquisition event with evidence for directionality of both G→M and M→G exchange. The top protein classes encoded by these genes were hypothetical (*n*=58), ribosomal (*n*=12) and membrane proteins (*n*=2).

Whilst these analyses confirm continuing sequence transfer, they do not address whether the exchanges occurred between individual NG–NM pairs or when these exchanges happened. Examining sequence variation around loci implicated in exchanges allows for inferences of timescales, with identical sequences indicative of more recent events prior to the accumulation of genetic variation. In contrast, non-identical sequences reflect older exchanges that have subsequently been shaped by mutations over longer evolutionary timeframes.

**Fig. 2.**
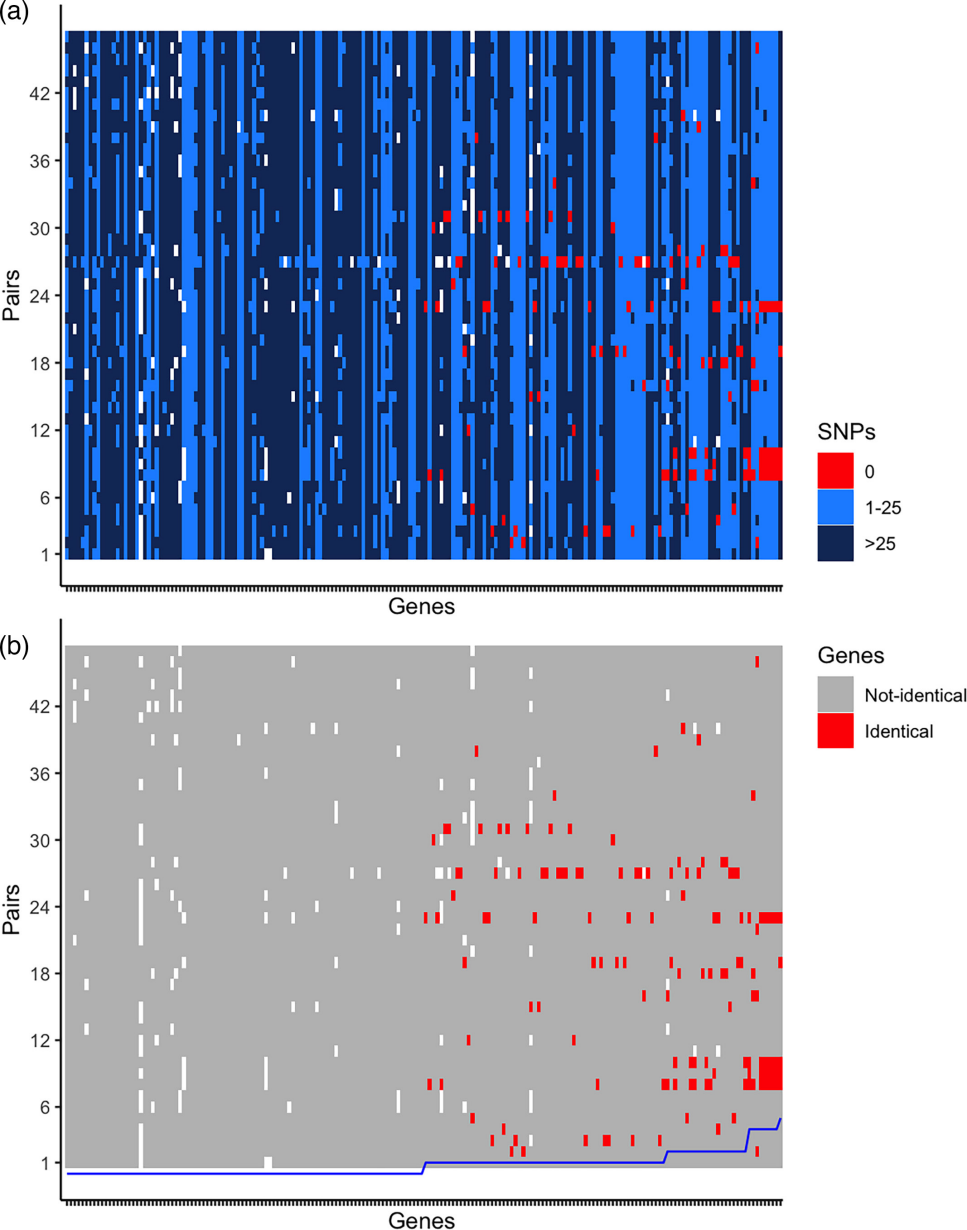
Gene diversity between isolate pairs. (a) The number of variant positions (i.e. Hamming distance and SNPs) between NG–NM pairs (pair identity on the y-axis) for 184 individual genes on the x-axis, in which there was evidence of inter-species exchanges (see text for details). (b) Sequence identity between NG–NM pairs on the y-axis for the same 184 genes on the x-axis. The blue line indicates the number of NG–NM pairs with an identical sequence, reflected in the ordering of the genes. Grey tiles indicate when the gene is present in both species in the pair, with red showing pair-identical sequences. In both panels, white regions indicate the absence of a gene in either or both the NM and NG isolate(s) of the NG–NM pair.

This analysis was limited to the 184 genes harbouring detected inter-species exchanges, across the 47 NG–NM pairs. Pairwise comparisons revealed a mean of 44 nucleotide variant SNPs (range 32-51) across these genes within a pair (Fig. 2a). For 94 genes, no identical sequences were detected between pairs, consistent with a past or distant transfer event. In the remaining 90 genes, an identical gene sequence (0 SNPs) was found in 1 to 5 pairs, consistent with more recent transfer events (Fig. 2b). Phylogenetically, isolates clustered with their pair and other isolates with the same gene allele (see Supplementary Table for tree patterns). Intriguingly, we observed that unencapsulated NM strains (122 events in 28 pairs) were more likely to be associated with pair exchange events than encapsulated strains (22 events in 19 pairs) (chi-squared *P*<0.001). In the majority of these events (85.9%), NMs were recipients of the gene from NG.

**Fig. 3.**
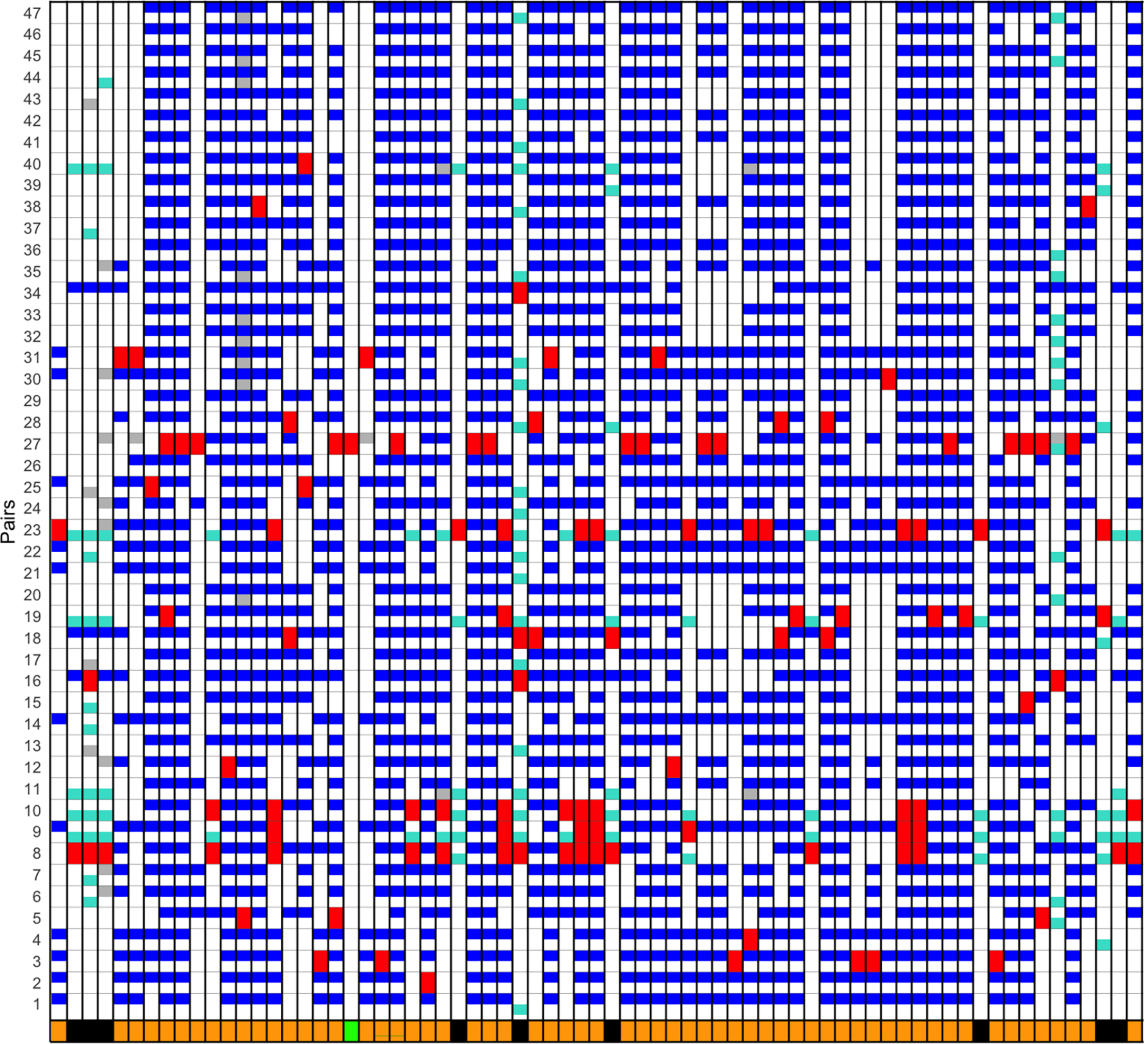
Gene sequence identity and exchange events between isolates. NG–NM pairs (upper: NG, lower: NM) are arranged on the y-axis, for 71 genes along the x-axis. The NG–NM pair involved in the gene exchange is represented by the paired red tiles along the y-axis. White and grey tiles indicate sequence divergence and absence of a gene, respectively. The blue (top) or turquoise (bottom) tiles in each paired row represent identical gene sequences to the exchanged sequence and represent potential alternative NG or NM donors in the dataset, respectively. The likely species origin of the gene allele is shown in the bar below the x-axis by black and orange tiles for NM and NG donors. The green tile indicates a single gene common to one NG–NM pair only with no alternative donors. See text for further details.

A possible alternative donor was sought for all genes with an identical sequence and exact PubMLST matches (*n*=76). To minimize false associations, the analysis was further restricted to genes for which complete sequence data were available for at least 40 pairs (*n*=71). A possible alternative donor was identified within the dataset for every identical sequence in an NM–NG pair, except for a single gene, NEIS0105 (indicated by the green bar in Fig. 3). The posterior probability that this event occurred during a period of co-carriage was estimated at 99.98%, based on a random draw of finding the matching NEIS0105 allele in either species, with 0.04% of NM (34 out of 87,314) and 11.4% of NG (4,624 out of 40,409) sequences containing this allele in the PubMLST genome database.

**Fig. 4.**
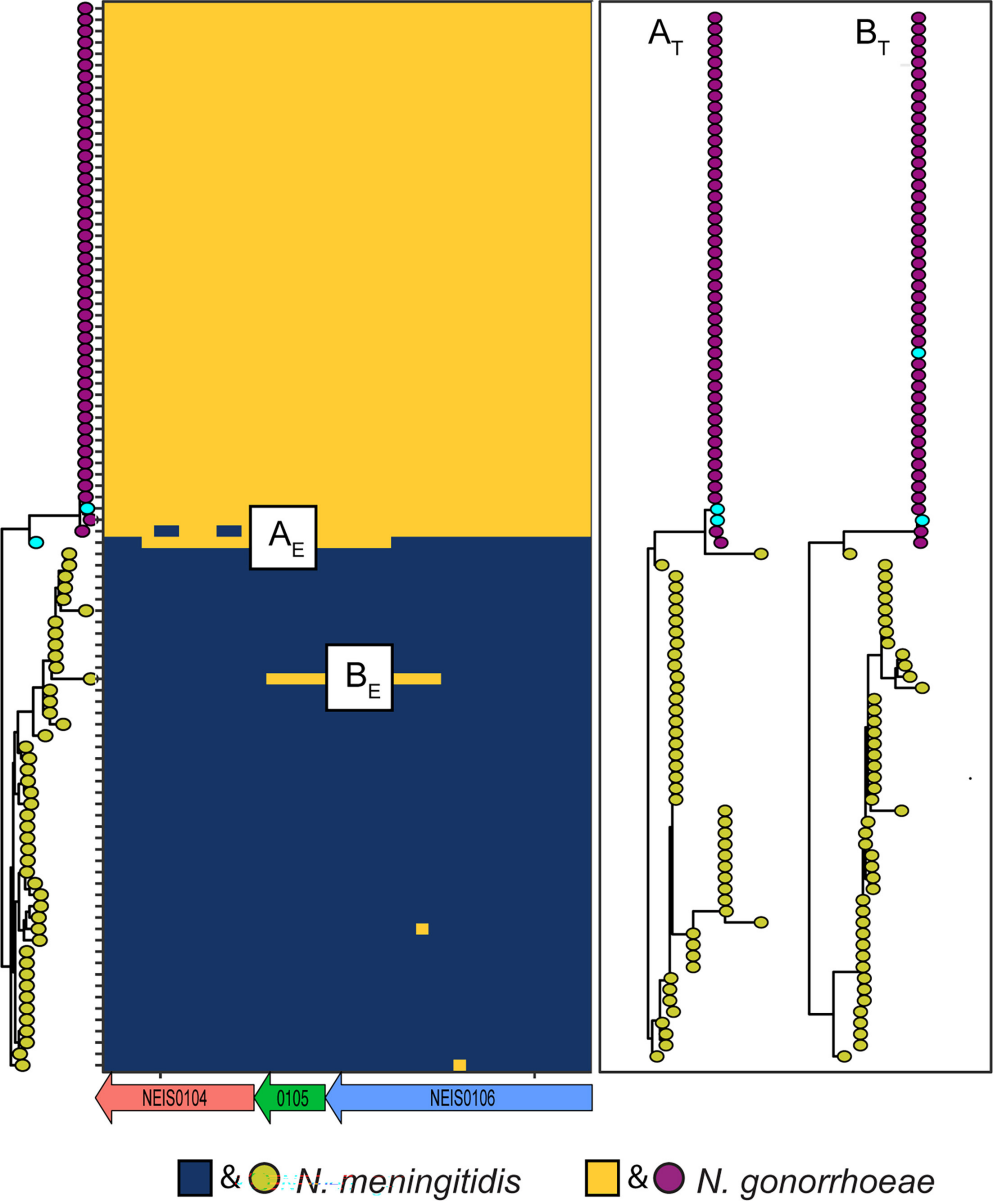
Recombination resulting in gene exchange between isolate pairs. Maximum-likelihood phylogeny of NM and NG for an 8,852 bp sequence fragment with species shown by coloured tip labels except for 1 NG–NM pair (cyan tips). The panel to the right of the tree reflects the first 3,452 bp of this fragment and consists of 3 genes (NEIS0104, NEIS0105 and NEIS0106) labelled below the x-axis. Colours along rows depict the corresponding genetic origin of a sequence, yellow for NG and blue for NM. Two exchange events >1,000 bp in length, labelled A_E_ and B_E_, are shown. The recombination event (A_E_) leads to a mosaic sequence and places the NM within the pair between the NG and NM groupings. The phylogeny, A_T_, in the second panel represents the sequence of event A_E_ and shows the expected side-by-side clustering of the pair. The second event, labelled B_E_, is noted with the corresponding phylogenetic tree (B_T_) and shows separation of the paired isolates (cyan) and therefore represents a more distant exchange compared to A_E_. Note that the pairs for events A_E_ and B_E_ are different.

Given that intra-pair transfer events are unlikely to be precisely restricted to single genes, we extended our analysis to adjacent sequences, with larger matching genomic regions increasing the perceived probability that each event is associated with the current episode of coincident NM–NG carriage. Analysis of the *de novo*–assembled contigs identified 15 collinear blocks spanning between 2 and 6 genes, with a median length of 4,939 bp (range: 684–8,808 bp). Using FastGEAR, two exchanges >1,000 bp [log(BF) >70] were detected in one of the collinear blocks. One exchange was noted across a 3,452 bp fragment encoding 3 genes: sasA (NEIS0104), an adaptive sensory response kinase; a hypothetical protein (NEIS0105); and a ribosomal small subunit protein (NEIS0106) (Fig. 4).

On examination of the above sequences, a gonococcal fragment (labelled A_E_) identical to the sequence of the NG pair has been captured by its NM counterpart, leading to the formation of a mosaic sequence, located between NG and NM clusters on the phylogeny. The tree for fragment A (labelled A_T_) alone shows the sequence identity within the co-isolated pair. An additional capture of NG sequence by NM (labelled B_E_) was identified in the same analysis. In this case, the paired sequences were not identical, likely ruling out a second transfer within the sampled coincident NM–NG pair. The calculated odds of finding this exact sequence configuration by chance were 166 million to 1 (a probability of 6.02×10^−9^) based on frequencies of finding the identical sequence in NG (4 out of 48,640) and NM (1 out of 13,659) within the NCBI database sequences, suggesting that this exchange event most likely occurred during the current episode of the coincident NM–NG carriage.

## Discussion

Analysis of consecutive pharyngeal swabs obtained from high-incidence populations attending an inner-city sexual health clinic over a 12-month period revealed an NG infection rate of 38%. This population undergoes routine screening for incident NG infections. Co-location of both pathogenic *Neisseria* species within the oropharyngeal niche was observed in an unexpectedly high proportion of individuals, with NM isolated in 29% of patients infected with NG. In contrast, NM prevalence from carriage studies in the general population is reported to be ~10% [1]. Of the NM isolated, 19 were groupable (serogroup B=15; X=2; W=1; E=1), and 28 were non-groupable. The presence of groupable NM isolates highlights the potential risk for invasive meningococcal disease in this population. Furthermore, the relatively high carriage rate may explain the documented IMD outbreaks in this setting.

An in-depth examination into the potential genetic exchange between these closely related, yet distinct, bacterial species was undertaken. Using a comprehensive approach to assess genetic exchange, we found several patterns indicative of varying timeframes for inter-species exchange events, emphasizing the complexity of the genetic exchange process. Notably, within the context of a larger DNA fragment, we identified one instance of unequivocal recent gene transfer between paired isolates occurring specifically between NM and NG in the pharynx of the individual sampled. Although such exchange events are infrequent, our findings have broader implications.

By identifying exchanges between NM and NG (copying and capturing genes), this study brings into focus the possibility of transfer of AMR determinants, especially to NM. The probability of AMR exchange is heightened by the unexpectedly high frequency of coincident NM–NG in the pharynx of high-incidence populations. Notably, several pilus genes, implicated in pathogenesis and colonization, were recently exchanged. Exchange rates were lower in encapsulated NM, which commonly causes invasive disease, compared to non-encapsulated NM [1]. Although encapsulated strains may be less prone to inter-species exchange, intra-NM exchange is widespread [26].

No instances of genetic exchanges through other commensal *Neisseria species* acting as an intermediary gene reservoir were observed. Although this mechanism is not excluded, a three-way exchange seems less likely to occur. Nevertheless, the role of other commensal *Neisseria species* should not be underestimated, as these have been documented as sequence donors leading to the formation of ceftriaxone-resistant *penA* alleles in NG [27].

The isolates originate from a single sexual health clinic collected from a high-incidence population. This population would be among the most likely to harbour resistant NG strains, and accordingly, NM resistance is more likely to emerge within this population. These findings challenge traditional microbiological approaches and highlight the importance of considering genetic dynamics in co-localizing pathogenic species for a more comprehensive understanding of their behaviour and potential clinical consequences, as well as revealing new opportunities for prevention.

In conclusion, this study contributes to the understanding of the intricacies of *Neisseria* evolution and adaptation. In pharyngeal swabs from high-incidence populations, coexistent NM and NG were more common than anticipated based on NM carriage estimates. Evidence of *inter*-species gene exchange over varying timeframes included an unequivocal signature of recent gene transfer between paired isolates. These findings provide new and valuable insights into the genetic exchange patterns between NM and NG and highlight the potential risks for AMR exchange in these pathogens.

## Supplementary material

10.1099/mgen.0.001623 Uncited Fig. S1.

10.1099/mgen.0.001623 Uncited Supplementary Material 1.

10.1099/mgen.0.001623 Uncited Supplementary Material 2.

## Abbreviations

AMR: antimicrobial resistance
NG: *Neisseria gonorrhoeae*
NM: *Neisseria meningitidis*
