# Supplementary material for: Gene exchange between Neisseria meningitidis and Neisseria gonorrhoeae
Source: Microb Genom. 2026 Jan 19;12(1):001623. doi: 10.1099/mgen.0.001623 (PMC12816886; doi:10.1099/mgen.0.001623)
Supplement: Uncited Fig. S1. [file mgen-12-01623-s001.pdf]

Supplementary Figure 1:

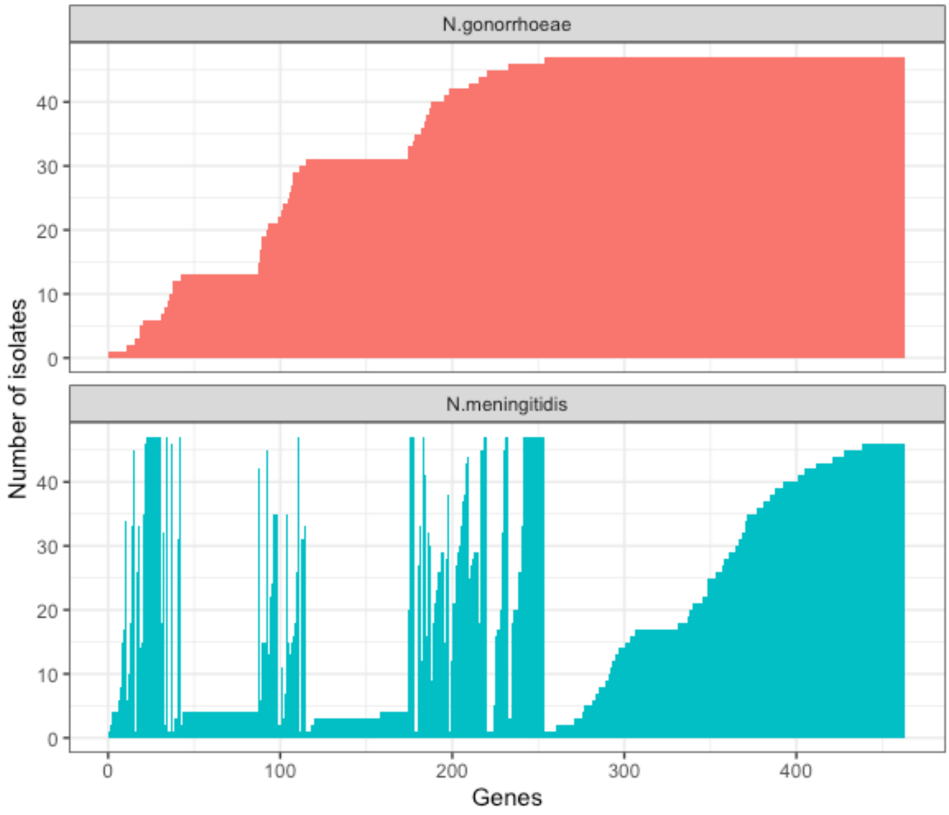

Pan-genome analysis: Pan-genome gene content by species for the 463 genes on the x-axis present in both species excluding the 1411 common genes present in all isolates.
